# Automatic Generation of Figural Analogies With the IMak Package

**DOI:** 10.3389/fpsyg.2018.01286

**Published:** 2018-08-06

**Authors:** Diego Blum, Heinz Holling

**Affiliations:** Chair of Statistics and Methods, Department of Psychology, Westfälische Wilhelms-Universität, Münster, Germany

**Keywords:** Automatic Item Generation, figural analogies, Item Maker, LLTM, rules

## Abstract

Automatic Item Generation (AIG) techniques are offering innovative ways to produce test items as they overcome many disadvantages involving standard item writing, such as time-consuming work and resource-intensive demands. Although this field is relatively new, it is progressing at a high speed, and several contributions have been accomplished. Nevertheless, a scarce amount of AIG software evidencing favorable psychometric properties of the generated items has been made accessible to the broad scientific community. This research had two goals: first, to present an empirical study of items produced with the aid of the Item Maker (IMak) package available online and, second, to present IMak itself for the automatic generation of figural analogies. We were particularly interested in assessing whether automatically created figural analogy rules could predict item psychometric difficulty. A total of 23 items were generated and administered to 307 participants, 49.51% from Germany. The mean age was 28.61 (*SD* = 10.19) and 57.65% of the participants were female. Results reveal adequate psychometric properties including convergent validity, that most of the manipulated rules contribute to item difficulty, and that rule-based difficulty prediction is possible to some extent. In other words, psychometric quality of the generated items is supported, which reveals the utility of the IMak package in assessment settings. Finally, the package is presented and its functions for figural analogy item generation are further described.

## Introduction

Many of the most well-known psychological and educational tests such as the Test of English as a Foreign Language are being administered every year (therefore, exposed) to large audiences, reason why their items need to be frequently renewed. Such activity of filling item banks on regular basis usually requires the conscious-controlled writing of items, task that is claimed to be resource intensive as well as time consuming. Therefore, new approaches to test design are being expanded. For example, the assessment engineering approach applies engineering-based principles and technology-enhanced processes to perform the design and development of tests (Lai et al., 2009; Gierl and Haladyna, 2013).

Computer-based Item Generation (better known as Automatic Item Generation, AIG) is a young but quickly evolving research area, and it consists of the computer-algorithm-controlled creation of items under a predefined item prototype called Item Model (IM; see, e.g., Lai et al., 2009; Gierl and Lai, 2012). With software as such, writing each individual item is no longer necessary. Instead, computer algorithms are utilized to generate families of items from a smaller set of parent IMs (Glas et al., 2010; Gierl and Lai, 2012). AIG strongly increases the number of items generated in the same amount of time that it takes for standard item writers. Parallel test forms are easily created through AIG in order to reduce overexposure of a single group of items, thus enhancing test security. AIG is also expected to produce items with a wide range of difficulty levels, avoid construction errors, and permit higher comparability of items due to a more systematic definition of the prototype (Irvine, 2002; Lai et al., 2009).

Automatic Item Generation usually requires two steps: First, an IM is created or derived from a good-measurement-quality pre-existing test item. An IM is a general explicit prototypical representation of the items to be generated. It incorporates the properties of the future items such as stem (i.e., formulation of the problem), response options, and auxiliary information required for the generation of items such as text, images, tables, diagrams, sound and/or video. Second, with the aid of computer algorithms, the elements of the stem and options of the IM are modified through multiple combinations in order to generate new items (Lai et al., 2009; Gierl and Lai, 2012). This means that other items are automatically created through variations of these elements. Furthermore, specifications can be introduced into the IM so that the program excludes illogical or unfitting items, similarities within a large subgroup of items, or particular combinations (Lai et al., 2009).

As far as ability tests are concerned, the construction of an IM can be enriched if it is based on a theory that predetermines the level of item psychometric difficulty (from now on: *β*), among other measurement characteristics. As a result, all automatically created items might acquire known psychometric properties from the moment they are built, regardless of what the analysis of empirical data determines (Irvine, 2002). In other words, item parameters could be predicted by a cognitive theory rather than calibrated (Embretson, 1999).

Let *radicals* (Irvine, 2002) be those structural elements that significantly affect item parameters (such as *β*) and provide the item with certain cognitive requirements. One or more radicals of the IM can be manipulated in order to produce parent IMs with different *β* levels. Each parent can then grow its own family by manipulating other elements that Irvine (2002) called *incidentals*. Incidentals are surface features that suffer random variations from item to item within the same family. Items that have the same structure of radicals and only differ in incidentals are usually labeled as *isomorphs* (Bejar, 2002) or *clones* (Embretson, 1999; Arendasy and Sommer, 2012). Item cloning can be essentially of two kinds: On the one hand, the IM may consist of an item with one or more open places, and cloning is done by filling each place with an element selected from a list of possibilities. On the other hand, the IM could be an intact item which is cloned by introducing transformations, for example changing the angle of an object of spatial ability tests (Glas and van der Linden, 2003). The variation of items’ surface characteristics should not significantly influence the testee’s responses. This is the reason why it is believed that incidentals produce only slight differences among the item parameters of the isomorphs.

From a cognitive perspective, rule-based item construction (Embretson, 1999; Freund et al., 2008; van der Linden, 2008) can play an important role in the manipulation of *β*, rules being elementary cognitive operations required to solve the item (Carpenter et al., 1990; Kubinger, 2008). Rule-based item construction consists of matching rule definition with item features during their generation, which then allows rules to act as the main radicals of items. Rules can be based on any cognitive theory, but analogical reasoning has historically been one of the most preferred cognitive constructs to work with in this respect. As a result, items measuring certain types of analogical reasoning, such as proportional analogies of the figural kind, are abundant in the psychometric literature, and many of them use specific rules as a problem-solving strategy (e.g., Cattell and Cattell, 1960; Carpenter et al., 1990; Wechsler, 1997; Raven et al., 1998; Brown et al., 2010).

When an incomplete proportional analogy of the form A:B::C:? is presented, a rule can be conceived as the implicit relation between A and B, which needs to be applied to the C element in order to know the missing D term, given that C and D are expected to relate to each other in a similar way as A and B (Bertling, 2012). Before beginning with item construction, it is important to clarify how a rule is going to be operationally defined; in other words, knowledge is required about which observed changes from A to B and, therefore, from C to D will be the indicators of such a rule. That said, the correct response to an item has to be a necessary result of applying all of the rules that play a role in the stem or main problem of the item. This lets several cognitive processes be implicated in finding a solution. For a thorough understanding of how rules take part in problem-solving strategies of figural items, see Carpenter et al. (1990), and Blum et al. (2015). In the work of Blum et al. (2016), for example, the following rules were manipulated to construct 2 × 2 figural matrix items aiming to measure analogical reasoning, where each item offers the possibility to apply a unique rule or group of rules to two solution pathways (i.e., A:B::C:D and A:C::B:D) in order to reach the same missing D element:

- Rotation rules of the main shape:
  (1) Clockwise rotation by 90° from A to B and by 45° from A to C.
  (2) Counterclockwise rotation by 90° from A to B and by 45° from A to C.
  (3) Clockwise rotation by 180° from A to B and by 135° from A to C.
- Rotation rules of the trapezium:
  (4) Clockwise rotation by 90° from A to B and by 45° from A to C.
  (5) Counterclockwise rotation by 90° from A to B and by 45° from A to C.
  (6) Clockwise rotation by 180° from A to B and by 135° from A to C.
- Other rules:
  (7) Main shape reflection by *x*-axis from A to B and by *y*-axis from A to C.
  (8) One line segment subtraction from A to B and from A to C.
  (9) One-edge dot movement from A to B and two-edge dot movement from A to C.

The aforementioned rules were based on five general rules, namely: main shape rotation, main shape reflection, trapezium rotation, line segment subtraction, and dot movement. It is therefore possible to think of a larger nine-rule set as well as a shorter five-rule set. Also, the three main shape rotation rules cannot be combined with each other or with reflection, and trapezium rules cannot be combined with each other either. These combinations result in the cancelation of a rule or in the generation of other rules than those intended by the designer. For example, combining a 90° clockwise rotation of the main shape with this same rotation counterclockwise produces no rotation as a result, and the *x*-axis reflection of the main shape combined with a 180° rotation of this shape part can be thought of as a *y*-axis reflection by its own.

Precedents of AIG of the figural kind made themselves available during the past 19 years with the works of Arendasy (Arendasy, 2005; Arendasy and Sommer, 2010; Arendasy et al., 2010), Freund et al. (2008) and Embretson and Reise (2000), among others. Figural item generators include MatrixDeveloper (Hofer, 2004), the Figural Matrices Generator GeomGen, and the Endless Loop Generator EsGen (Arendasy, 2002, 2005; Arendasy and Sommer, 2005). The first known item matrix generator was designed by Embretson (1998, 1999). As for our research team, rule-based item generators have also been created for statistical word problems (Holling et al., 2009; Holling et al., 2010). All of these generators were implemented to build items which demonstrated good psychometric properties, and in some cases, item psychometric difficulty (*β*) could even be predicted by a set of cognitive rules. Nevertheless, to the best of knowledge, these software tools are not yet extensively available to the worldwide scientific community, which makes it difficult to replicate the studies made by their authors.

In the present article, an empirical study of items created with the aid of the Item Maker (IMak) package, programmed by this paper’s first author (Blum, 2018) and freely available for R software environment (R Core Team, 2016), is presented and described. Throughout the paper, it will be particularly important to determine the extent to which IMak-generated figural analogy rules can predict item psychometric difficulty (*β*), since the more plausible this *β* prediction is, the more certain we can be about the utility of the IMak package in research psychology. Furthermore, as the aforementioned rules employed by Blum et al. (2016) have been used for the AIG process of the present research as well, this gives us the chance to compare both studies and to see whether rule contributions to *β* show stability over time. Finally, the IMak functions for figural analogy item generation are described. As this investigation deals with freely available software, forthcoming AIG studies may include further assessments of items created with IMak to substantiate the quality of such a package.

## Materials and Methods

### Sample

The study was headquartered at the Wesfälische Wilhelms-Universität (WWU) Münster in Germany and developed online from March 2016 until November 2017 through the platform Concerto v3.9.14 (Kosinski et al., 2012). A total of 307 participants who completed the scale were studied, 152 (49.51%) of whom were from Germany, 72 (23.45%) were from Indonesia, 11 (3.58%) were from Argentina, and the rest came from other countries. The mean age was 28.61 (*SD* = 10.19) and 57.65% of the participants were female. An important amount of German testees involved Psychology students of the WWU Münster who received course credits for participation after completing the test.

Each participant provided written informed consent. The study and consent procedure was approved by the Ethikkommission des Fachbereichs Psychologie und Sportwissenschaften of the Westfälische Wilhelms-Universität.

### Procedure

**FIGURE 1:**
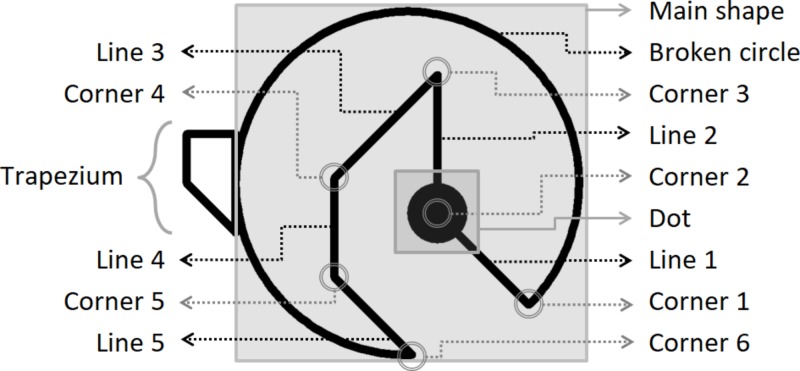
Basic shape used for item construction.

**FIGURE 2:**
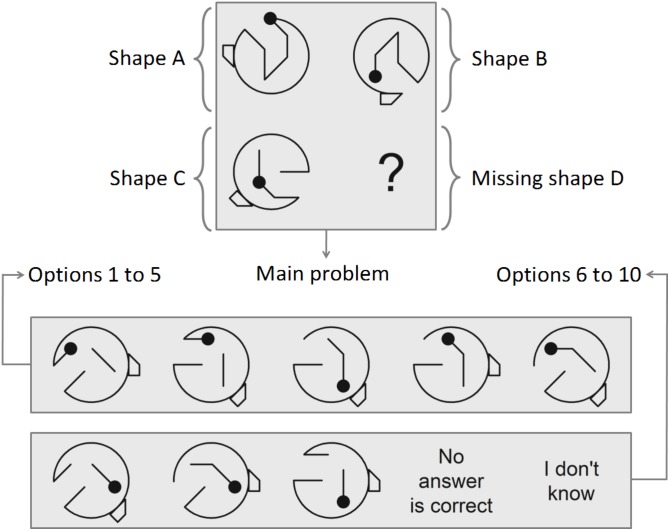
Plot mode “A” of a four-rule-based item.

The sample completed an online Test of Figural Analogies consisting of 23 items in counterbalanced order. Although more items could have been created for this purpose, we decided to be cautious and to use a limited amount to control possible fatigue effects. These items were automatically generated with the aid of the IMak package. For a graphical illustration of a basic shape used by IMak for item construction, see **Figure 1**. In this figure, lines are conceptualized as the internal line segments or edges of the main shape, whereas corners are the vertices connecting these segments. **Figure 2** shows an example of a four-rule-based item. Both figures delineate some properties to consider.

The properties of each of the automatically generated items resemble the ones described by Blum et al. (2016), and the purpose of such items is to measure figural analogical reasoning (Blum et al., 2011). The nine rules described in the introduction section of the present research were manipulated, and they were either used alone or combined during the AIG.

As previously mentioned, two solution pathways are possible given **Figure 2**, that is to say A:B::C:D and A:C::B:D. In either case, the reasoning is always meant to begin from A. While the rule(s) affect(s) how the analogical relation is going to be thought, the initial position each shape part adopts in shape A affects how this relation is going to look like. This is a key difference between structural (i.e., conceptual) relations and visual appearance. While the structure is given by radicals, which are the rules in this case, the visual state of shape A is given by incidentals.

The online assessment started with instructions during which testees had to find the solution to at least one practice item per general rule at a time (see “IMak on the field” in the Electronic **Supplementary Material** for more information). Practice items were easy to solve, as they only consisted of one rule. When individuals found the correct solution, a congratulations statement appeared, and they could move on to the next practice item. When they did not find the correct solution, a second practice item belonging to the same general rule was presented by the program, and the same statement was shown after finding the right answer. When individuals subsequently failed two practice items of the same general rule, instructions continued anyway without a concrete reply from the program.

Because it was an online study that they could complete in solitary, specific time limits were not provided, but we encouraged them to solve the whole test in one sitting without leaving part of it for another time. Also, item response times were automatically calculated. Individuals were told that they could use either of the solution pathways to arrive at a solution, and they were advised to go through the test on their own, that is to say, without receiving external help or being distracted.

### Data Analysis

Although 317 individuals completed the scale, six of them were eliminated because they responded ‘I don’t know’ in more than half of the items, and four more were also disregarded as they finished the test very quickly (less than percentile 1 of the total time spent in answering all of the items), which could have affected their results. Thus, a total of 307 protocols were chosen for further assessments.

In order to explore the psychometric quality of the automatically generated items, studies regarding reliability, convergent validity, unidimensionality, Rasch (1960/1980) model and Linear Logistic Test Model (LLTM; Fischer, 1973) fits are reported here. Data analyses were performed with the aid of R packages and with FACTOR (Lorenzo-Seva and Ferrando, 2018). Reliability indices included Cronbach’s α and the Greatest Lower Bound (GLB), the latter offering a more realistic lower bound of the true reliability than the former (Sijtsma, 2009). Convergent validity between total scores of this automatically generated test and the items used for the second study of Blum et al. (2016) was evaluated through Pearson’s *r* coefficient in 185 individuals of the sample.

Unidimensionality was assessed on the tetrachoric correlation matrix through two different methods: unrotated Principal Components Analysis (PCA) with the psych (Revelle, 2017) package, and unrotated Minimum Rank Factor Analysis (MRFA) with FACTOR. With respect to PCA, it should be shown that the first eigenvalue accounts for more than 40% of the total explained variance as strict criterion, and the quotient between the first and second eigenvalues should be higher than five (Carmines and Zeller, 1979; Martínez Arias, 1995); also, according to Cattell’s (1966) Scree Test, if the scale is meant to be unidimensional, then there should be an abrupt jump between the first and second eigenvalues, whereas a semi-horizontal line should be displayed for factors not accounting for meaningful variance. Moreover, the advantage of MRFA over PCA is that it can be used to calculate the explained common variance, which is the total common variance minus the unexplained common variance (Shapiro and ten Berge, 2002), thereby being a more stable index of unidimensionality. The number of dimensions of the factor model was forced to 1 for MRFA. The authors of this paper were looking forward to find a percentage of explained common variance of the first eigenvalue higher than 40% as well.

Rasch model item and test fits were studied with the eRm package (Mair et al., 2009) by using a conditional maximum likelihood approach for parameter estimation, thus following the same method as Blum et al. (2016). On item level, the Wald-type test (Glas and Verhelst, 1995) was employed. The statistical hypothesis here is that difficulty parameters are the same across subsamples. On test level, overall Rasch model fit was assessed with Andersen’s Likelihood Ratio (*LR*) test (Andersen, 1973). This test compares the item parameter estimates of different groups to the overall estimates by means of a conditional likelihood ratio statistic (Mair et al., 2009).

The LLTM was used to explore the impact of rules on item difficulty. The LLTM is an extension of the Rasch model, as it provides a linkage between item difficulty estimates and cognitive operations defined here as the rules. In an LLTM, model item difficulty parameters *β* are dependent on a linear combination of basic parameters *α*, which are in turn the difficulty estimates of the cognitive operations. The LLTM splits *β* into the following linear combination (Kubinger, 2008):

$${\text{β}}_{i}=\sum_{j=1}^{p}\omega_{ij}\alpha_{j}$$

where the *i*’th item difficulty parameter (*β_i_*) depends on the sum of products between the *j*’th basic parameter (*α_j_*) and its weight on the *i*’th item (*ω_ij_*) (Scheiblechner, 1972). In this sense, the *Q* matrix of the Electronic **Supplementary Material** shows the weight that each rule (therefore, each *α_j_*) has on each item of the test, with binary values determining weight presence or absence.

Traditional models were studied as in Blum et al. (2016) with the eRm package. Additionally, the lme4 (De Boeck et al., 2011; Bates et al., 2015) package was employed to fit generalized linear mixed-effects models considering person random effects (i.e., random-effects models). For both model types, two steps were accomplished. First, a Rasch model with 23 item predictors was fitted. Then, two LLTMs were fitted, one with five and another with nine rule predictors based on the five- and the nine-rule sets respectively (see Introduction). For each LLTM, once the basic parameters *α* were obtained, every given item parameter (*β_i_*) was calculated with the above-mentioned formula. Finally, with respect to random-effects models, the same LLTMs were fitted on the data of the 27 items administered to the old sample of 422 students (Blum et al., 2016).

With the purpose of studying rule-based item difficulty prediction, the following analyses were made on the current data. Rasch model and LLTM item difficulty parameters were plotted together in order to picture the possible linear relation between them. An outlier check was performed by making simple regressions of Rasch model item parameters on the LLTM ones and calculating Cook’s distances to assess the residual of each data point. The correlation between these sets of parameters, the determination coefficient and the test of hypothesis about the difference between model deviances, aimed to assess which of these models shows a better fit.

## Results

The test has a satisfactory reliability (Cronbach’s α = 0.89, GLB = 0.93). In fact, similar Cronbach’s α are usually reported in the literature of spatial ability tests; for example, Bertling (2012) reported α = 0.83 for his Figural Analogy Test as well as α = 0.81 for Gittler’s (1990) Three-dimensional Cube Test, and Freund et al. (2008) reported α = 0.93 for their Figural Matrix items. Moreover, Rasch model test fit is achieved with Andersen’s *LR* test (*LR*_22_ = 31.21, *p* = 0.09), and all items fit the model according to the Wald test (*p* > 0.01). A graphical inspection of the person parameters shows that person abilities are normally distributed. The inclusion of demographic variables (i.e., Gender and/or Secondary Education) in the Rasch regression model by means of the lme4 package does not alter item parameters greatly, and such variables display non-significant effects overall.

Furthermore, correlations were explored. The correlation between the total scores of the automatically generated scale and those of the eight items used for the second study of Blum et al. (2016) is 0.79 (*p* < 0.001). Item proportions of correct responses (IPCR), item mean response times (IMRT), and item rule amounts (IRA) can be found in the Electronic **Supplementary Material**. The correlations among these three variables are *r*_(IRA,IPCR)_ = -0.60, *r*_(IRA,IMRT)_ = 0.83, and *r*_(IPCR,IMRT)_ = -0.87. In other words, the rule-number increase is followed by the decrease of the amount of correct answers as well as by the increase of response times, which is the expected result. According to Mulholland et al. (1980), the increasing response latency and decreasing response accuracy could be explained by the increasing working memory load, which is caused by reasoning with multiple changes as well as with additional elements included in geometric problem-solving analogies. As the IMak package does not add more elements than the ones available for figural analogy plotting, the increasing working memory load might be attributed mostly to the manipulated changes (i.e., the rules).

Unidimensionality criteria are satisfied when working with the tetrachoric correlation matrix. For PCA analyses, the percentage of total explained variance accounted for by the first eigenvalue is 43.98 (higher than 40%), the quotient between the first and second eigenvalues is 7.24 (higher than 5), and an expected graphical representation of the eigenvalues can be appreciated through the Scree Test (see the Electronic **Supplementary Material**). For MRFA, the total observed variance is 23, the total common variance is 21.54, and the explained common variance of the first eigenvalue is 8.75 (40.63% of the total), which is the expected result.

**Table 1** shows the LLTM basic-parameter (*α*) estimates for both the old sample from 2016, who responded to non-computer-controlled generated items, and the new one with automatically generated items. As far as the latter sample is concerned, it can be seen that a great number of rules display significant parameters at 5%. Moreover, results are approximately stable throughout the samples, except for parameters belonging to trapezium rotations.

**Table 1:** Linear Logistic Test Model (LLTM) basic parameters of the nine specific rules, given the old (Blum et al., 2016) and the new (current) data.

Five general rules	Nine rules	Traditional LLTM		Random-effects LLTM	
		Old data	New data	Old data	New data
Main shape rotation	(1) Short and clockwise rotation	0.55 (0.09)^∗^	0.82 (0.10)^∗^	0.21 (0.08)^∗^	0.35 (0.09)^∗^
	(2) Short and counterclockwise rotation	1.28 (0.09)^∗^	1.55 (0.11)^∗^	0.75 (0.08)^∗^	1.16 (0.10)^∗^
	(3) Long and clockwise rotation	1.25 (0.08)^∗^	1.23 (0.12)^∗^	0.69 (0.07)^∗^	0.86 (0.12)^∗^
Trapezium rotation	(4) Short and clockwise rotation	1.27 (0.07)^∗^	0.36 (0.09)^∗^	0.92 (0.06)^∗^	0.30 (0.09)^∗^
	(5) Short and counterclockwise rotation	1.40 (0.07)^∗^	-0.26 (0.09)^∗^	1.03 (0.06)^∗^	-0.29 (0.08)^∗^
	(6) Long and clockwise rotation	1.61 (0.09)^∗^	0.11 (0.09)	1.10 (0.08)^∗^	-0.01 (0.09)
Reflection	(7) Reflection	1.02 (0.07)^∗^	1.14 (0.08)^∗^	0.50 (0.06)^∗^	0.76 (0.07)^∗^
Subtraction	(8) Subtraction	0.58 (0.08)^∗^	0.61 (0.06)^∗^	0.81 (0.07)^∗^	0.41 (0.06)^∗^
Dot movement	(9) Dot movement	0.30 (0.05)^∗^	0.36 (0.07)^∗^	0.10 (0.05)^∗^	0.13 (0.07)


*^∗^Significant at 0.05. Standard errors are given between parentheses.*

**FIGURE 3:**
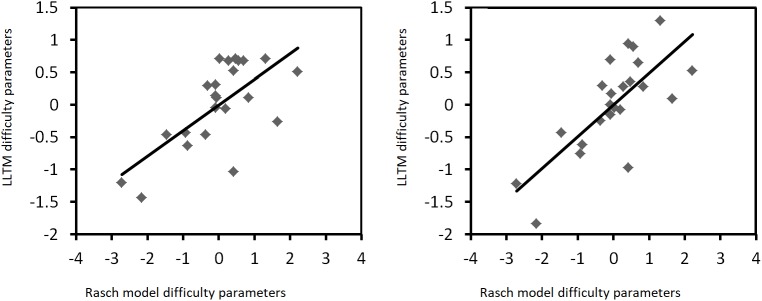
Rasch model-LLTM plots of mean-centered difficulty parameters when groups of five (left graphic) and nine (right graphic) rules are considered, based on traditional models.

**FIGURE 4:**
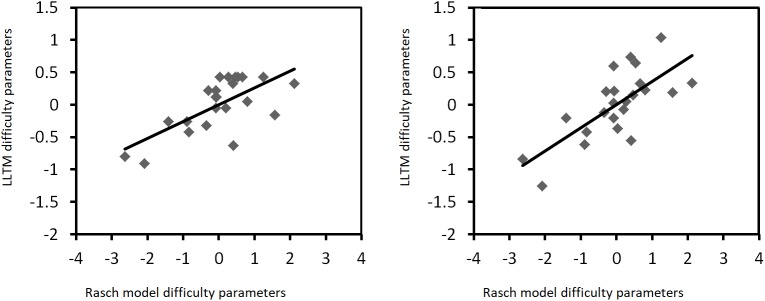
Rasch model-LLTM plots of mean-centered difficulty parameters when groups of five (left graphic) and nine (right graphic) rules are considered, based on random-effects models.

As for the relation between the Rasch model and LLTM estimates, correlation-coefficient results are presented in **Table 2**. Furthermore, Cook’s distances are not greater than 1; they do not even reach a value of 0.5, suggesting that none of the items is an outlier. **Figures 3, 4** represent the relation between the Rasch model and the LLTM item difficulty estimates when they are mean-centered. Correlation results are similar to those of other research performed with automatically generated visual-spatial intelligence items. For example, Freund et al. (2008) obtained *r* = 0.71 between Rasch model and LLTM item parameters of 25 Figural Matrix items; this means that a similar correlation with a similar item amount is evidenced in our study. Embretson (1999) regressed Rasch model item difficulties on item cognitive design principles of 36 Abstract Reasoning items, and obtained a multiple *r* = 0.77; in fact, “prediction levels comparable to a multiple correlation of at least 0.70 are usually obtained” (p. 410). With a much larger number of Mental Rotation items (*n* = 200), Arendasy and Sommer (2010) published a Rasch model-LLTM item difficulty correlation of 0.94.

**Table 2:** Coefficients of correlation (*r*), determination (*r*^2^) and adjusted determination (*r*^2^_Adj._) between the item difficulty parameters of the Rasch model and those of the LLTM for traditional models (TM) and random-effects models (REM).

	5 rules		9 rules	
	TM	REM	TM	REM
*r*	0.68	0.68	0.74	0.73
*r*^2^	0.46	0.47	0.55	0.53
*r*^2^_Adj._	0.44	0.44	0.53	0.50


According to the test of hypothesis about the difference between the Rasch model and LLTM deviances, two sets of results were obtained: With respect to traditional models, a significant difference (*p* < 0.001) exists both for the analysis with the five-rule (*LR*_17_ = 336.37) and the nine-rule (*LR*_13_ = 278.92) sets; the deviance of the Rasch model is lower than that of both the five- and the nine-rule-based LLTMs. As for random-effects models, a significant difference (*p* < 0.001) exists both for the analysis with the five-rule [*χ*^2^_(18)_ = 900.46] and the nine-rule [*χ*^2^_(14)_ = 772] sets; the Akaike Information Criterion (AIC) of the Rasch model is lower than that of both the five- and nine-rule-based LLTMs. In other words, the Rasch model fits better to the data. These results should be expected because, in general, the difficulty estimates of the LLTM do not equal those of the Rasch model, as the prediction will not be perfect (Wilson et al., 2008).

## Discussion

### Interpretation of Results

As explained in the introduction, freely available software tools for AIG with evidence of good item psychometric properties are rare nowadays. As a response, this paper intends to present results with respect to items automatically created with the IMak (Blum, 2018) package. Such results are far from being conclusive. As for that matter, larger samples as well as much larger item amounts should be studied. The accessibility of such a package might encourage researchers to further study its functionality and to perform complementary assessments. In this regard, we would like to decisively stress that future items created with the IMak package should not be used in applied settings as part of the working protocol without ensuring first that the items meet the required psychometric quality standards.

With respect to psychometric properties, the following can be argued. First, the 23 automatically generated items show favorable Cronbach’s α and GLB, as well as adequate test and item fits to the Rasch model; in other words, the figural analogy test exhibits good psychometric reliability in our study. As reliability was only assessed with this sample of examinees, it could change depending on the variance of the true scores. Second, the scale proves to measure one dominant construct as shown by the unidimensionality studies, the data correlates with that of other test measuring the same construct, cognitive rules that are indicators of such a construct make individual contributions to item difficulty, and most of these rule-based contributions are overall stable across studies; thus, the figural analogy scale shows acceptable psychometric validity in the present research as well. All in all, it can be said that the automatically generated items fulfill basic reliability and validity criteria, which is necessary for any psychological assessment context. Then again, it is important to remember that the aforementioned reliability and validity statements are limited to our study, and they should not be generalized yet.

As already mentioned, one specific interest regarding the study of the IMak-generated items was to assess whether item difficulty could be anticipated by means of a set of rules. As stated in the introduction, achieving this goal implies that difficulties can be predicted by a cognitive theory rather than calibrated (Embretson, 1999). A perfect difficulty prediction would suggest that, in theory, there is no need to put items to the test in order to realize how difficult they are; items with known difficulties could simply be created according to the demands of the research settings of interest, thus saving considerable time for psychometricians. As for our results, it can be argued that some degree of difficulty prediction is possible, because Rasch model item difficulty parameters share more than 50% of explained variance with the LLTM item difficulty estimates. But statistically speaking, the LLTM is not as good as the Rasch model at explaining such difficulty. Moreover, even though the graphics of **Figures 3, 4** seem to show a positive linear relation between both sets of item parameters, one can still notice that such graphical representations are not entirely homogeneous and linear. This is in part true because some items display a Rasch model-LLTM item-parameter relation that is rather different than that of the majority of items, and these specific cases could be interpreted as outliers from an intuitive point of view. All in all, item difficulty can be predicted to some extent by a set of automatically generated rules, meaning that a perfect psychometric difficulty prediction is far from possible. Furthermore, we might think that the latter interpretation is valid for other automatic generators of ability items and tests as well, as the prediction of all variance is very unlikely (Wilson et al., 2008; De Boeck et al., 2011).

Regarding rule-based contributions to item difficulty seen on **Table 1**, it is shown that main shape rotations, reflections, subtractions and dot movements are nearly consistent with respect to their individual contributions across samples. Taking trapezium rotations and subtractions aside, a steady order of difficulty (i.e., from the most to the least difficult rule) might be established: short counterclockwise main shape rotation, long clockwise main shape rotation, reflection, short clockwise main shape rotation, and dot movement. Particularly, the latter seems to make little or no contributions to item difficulty, reason why it should be studied further. Inconsistencies were only found with respect to trapezium rotations, as they were among the most difficult rules in the first study, but became very low difficulty predictors in the current assessment. This will be discussed below.

As for study limitations, it can be argued that the sample is somewhat heterogeneous with respect to age and country of origin, although most of the results presented here resemble those obtained in the former Blum et al. (2016) study. Furthermore, not only could results with respect to trapezium rotations not be reproduced across studies, but inconsistencies of our current data regarding these rotations also appeared, such as no influence on item difficulty (see rule 6 on **Table 1**) or even the opposite effect than the one expected (see rule 5 on the same Table). The reason for these irregularities is mostly unknown, but the following hypotheses could be established. First, as opposed to the items from 2016, the IMak package creates one more line in the interior of each basic shape (see **Figure 1** and compare it with the first two figures of Blum et al., 2016); maybe, the existence of this additional line gave testees more orientation points and helped them understand how trapezium rotations should be thought, thus reducing the difficulty of such a general rule. However, trapezium rotations are performed independently from the mentioned interior lines during AIG, and main shape rotations were apparently not affected by this line addition across studies, reasons why the described hypothesis might be discarded. Another hypothesis could rely on differences regarding test administration frameworks, with a paper-and-pencil version back in 2016 vs. an online test with respect to the current study; however, this still does not clarify why only one general rule has been affected. In any case, the data suggests that trapezium rotations should be further studied and/or treated with caution.

To summarize, the present research reveals favorable psychometric properties of automatically created items. In our study, the IMak-generated items have proven to measure analogical reasoning, as figural analogy rules made contributions to item difficulty. These items can also measure visual-spatial intelligence because, as explained in the results section, related batteries hold comparable psychometric properties. From a historical point of view, influential psychologists like Cattell (1971) and Spearman (1904) have used analogies to measure intelligence, as analogy items are among the ones with highest *g*-factor loadings (Sternberg, 1982). People showing brain injury, intellectual disability, aphasia and other similar conditions usually demonstrate difficulties to reason by analogy (Wolf Nelson and Gillespie, 1991), which reveals that analogical thinking is a key aspect of cognition, and that test batteries like the present one can be used for diagnostic purposes in clinical settings. Moreover, analogical reasoning is important for the cognitive development of crucial functions like abstract thinking, following a daily routine and solving complex problems (Wolf Nelson and Gillespie, 1991; Oliva Martínez, 2004), meaning that tests of this kind can be used for diagnoses in educational settings as well. Generally speaking, all applied settings in which the intellectual capacity needs to be put to the test can be benefitted from the analogical reasoning assessment, and the automatic generation of analogy items with measurement properties supported by empirical studies could help evaluating participants with items that are not repeated and for which anticipated psychometric characteristics might be established. However, it should be noticed that the results of the present research cannot be generalized yet, as it dealt with a reduced amount of rules, a short number of items, and only two samples were studied. Therefore, future research may involve further studies with the aid of the IMak package by assessing other generated items, varying the rules, or even taking samples from other populations. As already mentioned, a graphical inspection shows that person abilities are normally distributed, which is why we would not expect that the coefficients change greatly in other populations. But in order to be sure, more studies have to be made. For this reason, and to provide a thoughtful tool for item construction, the IMak package is explained below and in detail.

### The IMak Package

Package installation and use should be performed inside R Studio; for an easy introduction to package functionality, an R Studio template is provided as Electronic **Supplementary Material** of this paper. Item generation with the IMak package follows two steps, namely structure building and item shaping. These steps are represented by two distinct functions: build_fa and plot_fa. The following suggestions should be considered and compared to **Figures 1, 2** at all times for orientation purposes:

(1) Use the parameters of the build_fa function to specify the rules, and assign its output to an object. There are five general rules that can be manipulated with build_fa: main shape rotation, main shape reflection, trapezium rotation, line segment subtraction, and dot movement. Each general rule can be converted into a specific rule when the user gives precise arguments to their respective parameters. These parameters are the following according to the order in which the general rules were just mentioned: main.rot, mirror, trap.rot, subtract, and dot.mov. It should be remembered that main.rot and mirror are not allowed to be altered together inside the function. When applying a rotation rule, it is strongly recommended to work with two numerical values that are not so distant to one another (see the examples inside the package).
(2) Once the object is created with the build_fa function, use the plot_fa function to plot the information contained inside the object: plot_fa(object). If, at any time, you wish to save your items in a specific directory, then provide it between quotation marks in the following way: dir<- ”print here your directory”; plot_fa(object, directory = dir). Please do NOT save the plot in a directory by using the options provided by the R interface buttons; instead, the directory parameter inside plot_fa can do this effectively and straightforwardly. If info is not altered, then an additional CSV file is saved in the same folder where you save the items. The CSV file contains information about right answers to every item (given the order of options from left to right and top down) as well as the name of every general rule used.

A detailed description of the IMak functions for figural analogy item generation is provided as follows.

### The build_fa Function

This function generates the information of figural analogy items that can be read by the plot_fa function. It specifies the Item Model (IM) as well as the number of item clones. Although the build_fa function is able to work with a variety of arguments, only those belonging to radicals are compulsory and, therefore, at least one radical should always be manipulated by the user. Variables with their correspondent parameters available inside the function are the following:

• Number of isomorphs: The isomorphs parameter can be used to specify the number of items to be created within the same family. Even though the isomorph production can be unlimited, in particular cases the main problem of at least one isomorph may repeat the main problem of a previous isomorph after a certain item number, and this will be informed with a warning statement. For the case of all incidentals being left at random, this implies that, if the user wants to create more than 768 isomorphs, a warning message will emerge stating that items after number 768 may acquire the aforesaid repetition. The reason behind this is that a limited number of combinations among the possible positions of the constituent elements of shape A is available and, after the last combination has been reached, the function runs the same combination sequence again. Nevertheless, isomorphs can still be different from each other with respect to a randomized subtraction rule, the random placement of item options, or features that can be manipulated with the plot_fa function. The following examples illustrate how to create two isomorphs by means of one parent IM and four isomorphs by means of another parent IM:
two <- build_fa(isomorphs = 2, dot.mov = c(1, 2))
four <- build_fa(isomorphs = 4, main.rot = c(180, 135))
• Correct answer placement: The correct parameter can optionally be used to designate a set of possible locations for the correct response. Numbers from 1 to 9 are allowed as arguments, each belonging to a single option, the 9th one being the *no answer is correct* option. The function uses this set to select and build the right option of each isomorph in the provided spatial order. Such order must be read from left to right and top down in the cases of plot modes ”A” and ”B” (see **Figure 2**). Numbers are randomly chosen by default, and it is recommended to leave it like that. If items are meant to have high difficulty, it is strongly suggested to be cautious when the number 9 option is selected as the right one, due to reliability problems that may occur according to Blum et al. (2016). No matter the case, correct responses of every isomorph can be consulted with the plot_fa function. The following example shows how to place the right answer in position 1:
right1 <- build_fa(mirror = 1, correct = 1)
• Radicals: Radical parameters can be conceived as the IMak version of the general rules, whereas arguments that are set for these parameters designate the specific rules to work with. Such rules affect the item when at least one of the argument values does not equal 0. Changes of shape A to become B (i.e., A → B) and to become C (i.e., A → C) are manipulated together to affect both solution pathways at the same time, and they are designated by the first and second numbers of the argument respectively. An exception is made for mirror because a single number designates both changes. For the case of rotations, it should be remembered that positive numbers produce counterclockwise movements while the opposite happens with negative numbers, and values that are multiples of 45 and range between -135 and 180 are allowed. Furthermore, even though five radical parameters are available inside the function, mirror and main.rot cannot be manipulated together due to rule confusion as explained in this paper, which is why an error message is returned to prevent this action. This means that a maximum of four radicals can be manipulated at the same time. Radical parameters are the following:
(1) main.rot: It stands for Main Shape Rotation. Its argument designates the rotation angle of the main shape. Different rotation angles can be chosen for A → B and for A → C by creating a two-number vector. As an example, for the generation of rule 2 of the described research, main shape A is expected to rotate 90° counterclockwise to obtain B and 45° counterclockwise to obtain C; therefore, this rule can be automatically generated with the following argument: main.rot = c(90, 45).
(2) mirror: It stands for Mirroring or Reflection. Its argument designates whether a reflection relation between shapes should be present or not. The user can make a reflection by setting mirror to 1; as a result, main shape A performs an *x*-axis reflection to become B and a *y*-axis reflection to become C.
(3) trap.rot: It stands for Trapezium Rotation. Its argument designates the rotation angle of the trapezium. Different rotation angles can be chosen for A → B and for A → C by creating a two-number vector. As an example, for the generation of rule 6 of the present research, trapezium A is expected to rotate 180° to obtain B and 135° clockwise to obtain C; therefore, the correspondent argument should read: trap.rot = c(180, -135).
(4) subtract: It stands for Subtraction. Its argument designates one internal line segment of the main shape (from now on: line) per solution pathway to be subtracted. Different subtractions can be chosen for A → B and for A → C by creating a two-number vector. Given that there are five lines, the line number is a value within the 1:5 vector. For example, the argument may read: subtract = c(1, 4). Nevertheless, the easiest and most recommended way to apply subtraction is by leaving this line choice at random in the following way: subtract = ”R”.
(5) dot.mov: It stands for Dot Edge Movement. Its argument designates the number of edges (i.e., adjacent lines) the dot moves through in a particular direction until reaching a corner. One thing to keep in mind is that the dot *never* moves through the broken circle or the trapezium. Different numbers can be chosen for A → B and for A → C by creating a two-number vector. Since the dot can move through a maximum of five edges, the sum between the two numbers must be five at most. For example, an argument designating a total of three edges for dot movement may read: dot.mov = c(1, 2). The user must consider that, if the sum of both numbers is higher than 3, then the dot position of shape A is usually relocated towards the corners attached to the broken circle. This is done to give the dot more edges to wander through in a particular direction.
• Incidentals: They affect the position of the elements of shape A. They are randomly assigned by default, and they should only be manipulated when specific interests in their control arise. Out of the following parameters, a.main, a.trap and a.dot allow arguments of length greater than 1, thus giving the program the chance to select, for each isomorph, one value from a set of existing possibilities. Incidental parameters are the following:
(1) a.main: Its argument designates the rotated state of the main shape. Single values from 1 to 8 can be chosen. For example, the argument may read: a.main = 1.
(2) a.flip: Its argument designates whether the main shape is presented as flipped in relation to an axis or not, with logical values of T and F for the respective choices. For example: a.flip = F.
(3) a.trap: Its argument designates the rotated state of the trapezium. Single values from 1 to 8 can be chosen. For example: a.trap = 2.
(4) a.dot: Its argument designates the corner number for dot placement. Single values from 1 to 6 can be chosen, and they are sometimes automatically relocated when the sum of the dot.mov vector is higher than 3 (see dot.mov). For example: a.dot = 6.
(5) constrict: Its argument designates a part of shape A to display all possible positions every *n* isomorphs. A constriction can be helpful when willing to create a small amount of isomorphs and making sure at the same time that all of them are different to one another with respect to the part being constricted. Only one of the following arguments can be chosen: ”main” (for the main shape), ”trap” (for the trapezium) or ”dot”. For example: constrict = ”main”.
• Options: Radical parameters that use the al. prefix can be utilized to designate alternative (incorrect) solutions for each of the rules throughout the options by following a Solutions Combination Design (*SCD*; Blum et al., 2015, 2016). This means that for every rule intended to be manipulated in the item, a correct solution as well as a number of incorrect solutions are generated, and then solutions are combined across the rules to build the options. Each possible combination is a potential option, and the option that holds all correct solutions is the only right answer. The following *SCD*s are generated by default depending on the amount of combined rules within each isomorph:
- An *SCD* = 4 × 2 for one-rule-based items (i.e., four solutions for one rule and two for an extra rule, where the latter rule comprising two solutions is not present in the main problem),
- An *SCD* = 3 × 3 for two-rule-based items (i.e., three solutions for each rule, where one of the nine combinations not being the right answer is eliminated),
- An *SCD* = 2^3^ for three-rule-based items (i.e., two solutions for each rule), and
- An *SCD* = 2^4^ for four-rule-based items (i.e., two solutions for each rule, where combinations which hold three incorrect solutions or three correct solutions are eliminated to establish a more balanced presentation between correct and incorrect solutions).
As for items having the *no answer is correct* option as the right answer, the option containing all correct solutions is replaced by a similar option, providing that it does not repeat an existing one, or by one of the combinations that were targeted for elimination.
It should be remembered that alternative rotations use the right answer as the reference to rotate the shapes, and something similar happens with the alternative reflection. The same groups of values as the ones described for the radical parameters are allowed to be chosen. Alternative solutions are randomly assigned by default, which is recommended, but if the user has a specific interest in their control, then two actions should take place. First, automatic must be set to F as logical argument. Second, alternative solution values must be chosen for the applied rules by means of the following parameters:
(1) al.main.rot: Its argument designates alternative rotation angles for the main shape.
(2) al.mirror: Its argument designates alternative main shape positions, where both rotation and flipping can be combined to obtain different kinds of mirrored shapes. Each alternative solution requires the specification of two numbers, the first one containing a main shape rotation angle and the other stating whether reflection should be applied with respect to an axis (1) or not (0).
(3) al.trap.rot: Its argument designates alternative rotation angles for the trapezium.
(4) al.subtract: Its argument designates a maximum of two lines per alternative solution to be removed, providing that at least one of the corresponding line numbers differs from any of the numbers of the subtract argument. Every alternative solution comprises two numbers, each designating any of the lines from 1 to 5 or none (0).
(5) al.dot.mov: Its argument designates alternative corners for dot placement. Values from 1 to 6 can be selected. When the value of this argument coincides with the dot placement of the right answer, the alternative dot position is automatically relocated.
When alternative solutions are being chosen by the user, special care must be paid according to the number of rules, to select:
- Three alternative solutions for the rule applied in the main problem when working with only one rule. If add.rule is not altered, a randomly selected additional rule affects the options together with the main rule, which is the reason why the user must also select one alternative solution for each of the other rules. If the add.rule argument calls one specific rule from 1 to 5 (same order of the radical parameters), then the latter statement is only valid for this rule. If add.rule equals -1 and the user is only working with one rule, no extra rule is added but some limitations will be faced, normally stated through warnings and/or error messages.
- Two alternative solutions should be chosen for each rule of two-rule-based items.
- One alternative solution should be chosen for each rule of items based on more than two rules.

### The plot_fa Function

This function plots the information of figural analogies generated with function build_fa. It only works with objects of class fa_items created by the latter function. The output of the plot_fa function should not be assigned to an object, except when willing to use the optional parameters switch.from and switch.to. Unless other specific interests arise, the easiest way to plot isomorphs is by only naming the object of class fa_items inside the function. For further usage of the items in psychological assessments, it is recommended to save plots in a directory with the aid of the directory parameter. In other words, do NOT use other R tools to save items outside the platform; the plot_fa function can do this efficiently and straightforwardly when a directory argument is provided as input. The following variables can be manipulated, for which specific parameters are available:

• Information source: The first step the plot_fa function will make is to look for an object of class fa_items to work with. The object name should be provided as first argument (or as argument for items). It contains the source of information for isomorph plotting with no default. The following shows how to plot the R objects already given as examples for the build_fa function:
plot_fa(two); plot_fa(four); plot_fa(right1)
• Items to plot: The which parameter can be used to designate specific numbers of isomorphs to be plotted. These numbers must be within the amount of existing isomorphs. All isomorphs are plotted by default. When willing to use switch.from and switch.to, the isomorph number to work on must be specified as an argument for which, even if there is only one isomorph available (for the latter case, which should equal 1). For example:
plot_fa(four, which = 2:4)
• Plot mode: The argument specified for mode designates the arrangement of shapes when being plotted. Modes ”A”, ”B” and ”C” are available. Plot mode ”A” is chosen by default since it has been used for the presented research, thereby having some empirical support. For example:
plot_fa(two, mode = ”B”)
• Language for verbal options: Arguments specified for language and language.dir designate a language for options reading *no answer is correct* and *I don’t know*. The languages currently available are English (”E”), German (”D”), and Spanish (”S”), but English is a default choice for language, while all languages are chosen by default for language.dir. The difference between language and language.dir is that the former is used to choose the preferred language of isomorphs plotted inside the R interface, whereas the latter does this for the PNG files when a directory argument is provided by the user (see directory). For example:
plot_fa(two, language = ”D”)
• Form type: Arguments specified for form.int and form.ext designate, respectively, how the line segments of the main shape and those of the trapezium are arranged when being plotted. Forms of type ”A”, ”B”, ”C” and ”D” are available. Form type ”A” is chosen by default for both cases since it has been used for the present research, thereby having some empirical support. For example:
plot_fa(right1, form.int = ”B”, form.ext = ”C”)
• Directory to save isomorphs: The argument specified for directory designates a folder in your PC to store the isomorphs as PNG files. Make sure to provide such an argument between quotation marks and that the target folder exists.
• Information about isomorphs: When info is set to T by default, the function prints the relevant information about general rules applied as well as the correct answer to each isomorph. Additionally, when a directory argument is passed to the function, the mentioned information is saved in this directory as a CSV file.
• Two item options to switch with regard to their location: Arguments specified for switch.from and switch.to designate item options to be exchanged to each other’s positions. Potential arguments lie within the 1:8 vector, which specify positions of options that should be read from left to right and top down in the cases of plot modes ”A” and ”B” (see **Figure 2**). When willing to use these optional parameters, the output of the plot_fa function should be assigned to an object comprising the same name as the object of class fa_items used as an argument inside the function, so that the new changes can be saved into the latter object. The argument specified for which must designate the isomorph to be affected, even if there is only one isomorph available. See the package instructions for more details on this matter.

## Author Contributions

DB contributed conception and design of the study, including item generation, database confection, statistical analyses, and first manuscript draft. HH contributed to manuscript structure and content modification, as well as supplementary statistical approaches. All authors contributed to manuscript revision, read and approved the submitted version. DB takes primary responsibility for communication with the journal and editorial office during the submission process, throughout peer review and during publication. DB is also responsible for ensuring that the submission adheres to all journal requirements including, but not exclusive to, details of authorship, study ethics and ethics approval, clinical trial registration documents and conflict of interest declaration. DB should also be available post-publication to respond to any queries or critiques.

## Conflict of Interest Statement

The authors declare that the research was conducted in the absence of any commercial or financial relationships that could be construed as a potential conflict of interest.
