# Supplementary material for: Automatic Generation of Figural Analogies With the IMak Package
Source: Front Psychol. 2018 Aug 6;9:1286. doi: 10.3389/fpsyg.2018.01286 (PMC6087760; doi:10.3389/fpsyg.2018.01286)
Supplement: Supplementary file 1 [file Data_Sheet_1.ZIP › Few examples of IMak items.pdf]

Few examples of IMak-generated items

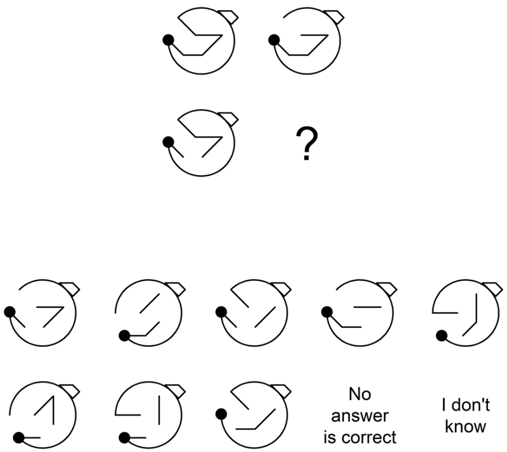

A. Item with subtraction rule plotted with default values.

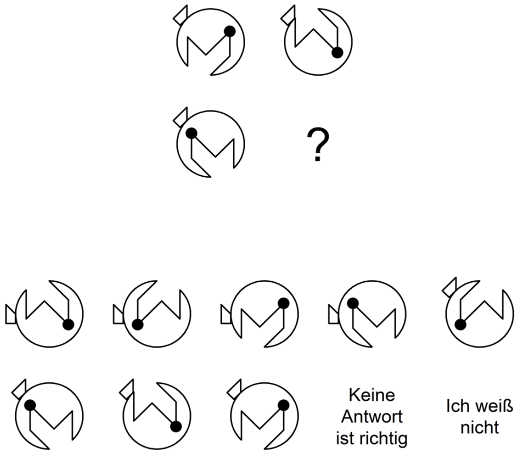

B. Item with reflection rule and shape form changes, plus German language.

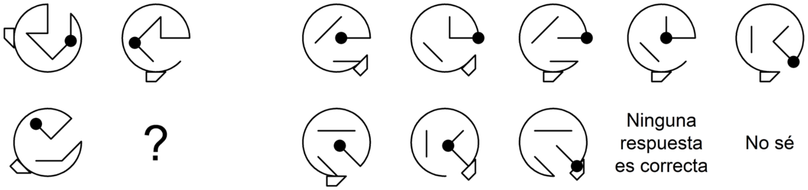

C. Item with four rules, plot mode and shape form changes, plus Spanish language.
