# Supplementary material for: Automatic Generation of Figural Analogies With the IMak Package
Source: Front Psychol. 2018 Aug 6;9:1286. doi: 10.3389/fpsyg.2018.01286 (PMC6087760; doi:10.3389/fpsyg.2018.01286)
Supplement: Supplementary file 1 [file Data_Sheet_1.ZIP › Q matrix of the IMak-generated items.pdf]

General rules (5 rules)					
Items	1	2	3	4	5
1	1				
2			1	1	
3	1			1	1
4		1	1	1	1
5		1			
6	1				1
7		1	1	1	
8	1	1		1	1
9			1		
10			1		1
11	1	1		1	
12	1	1		1	1
13				1	
14	1			1	
15		1	1		1
16		1	1	1	1
17					1
18	1	1			
19		1		1	1
20		1	1	1	1
21				1	1
22			1	1	1
23	1	1		1	1

Specific rules (9 rules)									
Items	1	2	3	4	5	6	7	8	9
1	1								
2							1	1	
3		1						1	1
4				1			1	1	1
5					1				
6			1						1
7						1	1	1	
8		1		1				1	1
9							1		
10							1		1
11		1				1		1	
12			1		1			1	1
13								1	
14	1							1	
15				1			1		1
16					1		1	1	1
17									1
18		1				1			
19				1				1	1
20						1	1	1	1
21								1	1
22							1	1	1
23	1				1			1	1
