# Supplementary material for: Automatic Generation of Figural Analogies With the IMak Package
Source: Front Psychol. 2018 Aug 6;9:1286. doi: 10.3389/fpsyg.2018.01286 (PMC6087760; doi:10.3389/fpsyg.2018.01286)
Supplement: Supplementary file 1 [file Data_Sheet_1.ZIP › IMak on the field.pdf]

IMak on the field

Case 1

A group of researchers wants to develop between 35 and 40 figural analogy items only comprising rotation rules, for which they require our help. We think that the R package IMak could serve to answer their needs. We already know that two distinct rotations can be manipulated with the `build_fa` function: main shape rotations and trapezium rotations. We also know that each of them can be used alone or they can be combined together in a single item. What shall we do then? We should not jump directly into item generation, but instead think in advance of a design by following the next steps:

1. We specify the values of several rules: For this matter, it must be remembered that we want to manipulate a certain change from shape A to shape B, as well as from shape A to shape C, to build the main problem of the item. The change from A to B is given by the first number of the argument, whereas the change from A to C is given by the second one, except for reflections where only one number designates both changes. If we work in the same way as the described research, then the following pairs of values can be proposed:
    a. Clockwise rotation by 90° from A to B and by 45° from A to C. In R code:
    ```
    rot1 <- c(-90, -45)
    ```
    b. Counterclockwise rotation by 90° from A to B and by 45° from A to C. In R code:
    ```
    rot2 <- c(90, 45)
    ```
    c. Clockwise rotation by 180° from A to B and by 135° from A to C. In R code:
    ```
    rot3 <- c(180, -135)
    ```
2. We assign these pairs of values to parameters `main.rot` and `trap.rot` of the `build_fa` function to build the rules:
    ```
    main.rot = rot1
    main.rot = rot2
    main.rot = rot3
    trap.rot = rot1
    trap.rot = rot2
    trap.rot = rot3
    ```
    Each of the six described rules can be used solely for the creation of an item (i.e., without making rule combinations), therefore giving a total of six items so far. For items based on combinations of both main shape and trapezium rotations, other six possibilities are the following:
    ```
    main.rot = rot1, trap.rot = rot2
    main.rot = rot2, trap.rot = rot3
    main.rot = rot1, trap.rot = rot3
    main.rot = rot2, trap.rot = rot1
    main.rot = rot3, trap.rot = rot2
    main.rot = rot3, trap.rot = rot1
    ```
    We do not create items where the main shape and the trapezium rotate in an equal manner because it would look like one unique rotation.
3. We create isomorphs to fill the remaining items: Researchers are requesting between 35 and 40 items, and we have until now only 12 prospective items. These can be thought of as 12 Item Models (IM) since they are different from one

another on the basis of rules, and each of them can potentially grow its own family of isomorphs by just altering their incidentals. Hence, to follow the researchers' request, it would be appropriate to create three isomorphs per IM to provide a total of 36 items.

To summarize, our design reaches 36 items of pure mental rotation strategies. Now it is time to write the R code and to give some directories to store all items in different folders. You may try the following code, and remember that target folders as well as a route to access them should also be included:

```
# Building the information:
rot1 <- c(-90, -45)
rot2 <- c(90, 45)
rot3 <- c(180, -135)
group1 <- build_fa(isomorphs = 3, main.rot = rot1)
group2 <- build_fa(isomorphs = 3, main.rot = rot2)
group3 <- build_fa(isomorphs = 3, main.rot = rot3)
group4 <- build_fa(isomorphs = 3, trap.rot = rot1)
group5 <- build_fa(isomorphs = 3, trap.rot = rot2)
group6 <- build_fa(isomorphs = 3, trap.rot = rot3)
group7 <- build_fa(isomorphs = 3, main.rot = rot1, trap.rot = rot2)
group8 <- build_fa(isomorphs = 3, main.rot = rot2, trap.rot = rot3)
group9 <- build_fa(isomorphs = 3, main.rot = rot1, trap.rot = rot3)
group10 <- build_fa(isomorphs = 3, main.rot = rot2, trap.rot = rot1)
group11 <- build_fa(isomorphs = 3, main.rot = rot3, trap.rot = rot2)
group12 <- build_fa(isomorphs = 3, main.rot = rot3, trap.rot = rot1)

# Create 12 folders with names: "group1", …, "group12" in your PC.

# Generating an object with the route to all these folders:
route <- "C:/Desktop"   # You might want to provide another route.

# Plotting the information and saving it into the folders:
for (i in 1:12) {
  plot_fa(get(paste0("group", i)),
          directory = paste0(route, "/group", i),
          language.dir = "E")
}
```

Case 2

Researchers are requesting 12 figural analogy items of high difficulty, to be administered online. They specifically require that the basic shapes of each item are submitted in separate files, since researchers need to upload them individually in order to make it easier to spot right and wrong answers with their code. We choose IMak to answer their request. We already know from our research that the number of rules has an impact on item complexity, because items with higher rule amount tend to be more difficult to solve. Therefore, in order to fulfill the researchers' needs, we would like to manipulate the biggest amount of rules possible simultaneously. After reading the present research and the one from Blum and cols. (2016), we can reasonably assume that, by constructing four-rule-based items (the biggest rule amount IMak can handle), we will be

providing items with a high difficulty level[1]. Also, how we specify each rule will have a decisive impact on difficulty. Having all this in mind, the design could be proposed in the following way:

1. We specify the rule values:
   ```
   rot1 <- c(90, 45)
   rot2 <- c(-135, -90)
   rot3 <- c(180, 135)
   mirror <- 1
   subtract <- "R"
   dot <- c(1, 2)
   ```
2. We assign these values to parameters `main.rot`, `mirror`, `trap.rot`, `subtract` and `dot.mov` to build the rules: We have configured three pairs of rotation values, which will be assigned to each of the two general rotations (i.e., main shape rotations and trapezium rotations), thus giving a total of six rules so far. Furthermore, values specified for reflection, subtraction and dot movement can be assigned individually, thereby configuring three more rules. This means that a total of nine rules are being thought:
   ```
   main.rot = rot1
   main.rot = rot2
   main.rot = rot3
   trap.rot = rot1
   trap.rot = rot2
   trap.rot = rot3
   mirror = mirror
   subtract = subtract
   dot.mov = dot
   ```
   The fact of having built more rotation rules than other rules is not related to an order of importance, but to the manipulation characteristics of each general rule inside IMak. For example, the reflection can only comprise one of two possible values according to its presence (1) or absence (0) in the main problem of the item, so no other specification is possible. Plus, the subtraction can be left at random, because we are assuming that any randomly subtracted line affects the testee's decision making in a similar way.
3. We make the combinations, each resulting in a four-rule-based item: We remember that rotations of the same attribute of the shape cannot be combined among each other, nor can main shape rotations be combined with reflection. Having this in mind, the following two designs are proposed:
   a. Main shape rotations combined with trapezium rotations, subtraction and dot movement. This gives a design of 3 × 3 × 1 × 1 = 9 four-rule-based items.
   b. Reflection combined with trapezium rotations, subtraction and dot movement. This gives a design of 1 × 3 × 1 × 1 = 3 four-rule-based items.

A total of 12 items results from the stated combinations, and 12 items are required by the researchers, so our initial thought would be that we have what they need so far. However, the designer might think that main shape rotations are being overrepresented here, reason why (s)he prefers to eliminate three of the first nine combinations

[1] Remember though, that studies still need to be accomplished as to whether this statement can also be confirmed for trapezium rotations.

(preferably those in which the trapezium rotates in an equal manner as the main shape), as well as to add one isomorph for each combination comprising reflection, thus giving a total of 12 items split in six main shape rotations and six reflections. The latter choice seems more balanced than the previous one.

Furthermore, given that all reflection rules are basically thought in the same way (i.e., the mirror axes that are utilized to reflect the shape are always the same for each pathway), the designer might be interested in making sure that the rotated state of every main shape A is not repeated across item families because, when it is repeated, then the function may generate reflections looking pretty much alike. We should then tell the program that, for each item family, a different set of possible positions of some A shape parts is allowed to be used. Since there are three Item Models (IM) for the reflection rule in our chosen design, we could tell the first IM to only allow main shape A positions from 1 to 3, the second one from 4 to 6 and for the third one we allow positions 7 and 8. A `constrict` argument could also complement this process within each item family for a more specific approach, but we will not deal with this in the code that is seen below.

Considering all things together, now it is time to write the respective R code. You may try the following in R, remembering also to include target folders as well as a route to access them:

```
# Building the information:
rot1 <- c(90, 45)
rot2 <- c(-135, -90)
rot3 <- c(180, 135)
mirror <- 1
subtract <- "R"
dot <- c(1, 2)
i1 <- build_fa(main.rot = rot1, trap.rot = rot2,
               subtract = subtract, dot.mov = dot)
i2 <- build_fa(main.rot = rot2, trap.rot = rot3,
               subtract = subtract, dot.mov = dot)
i3 <- build_fa(main.rot = rot1, trap.rot = rot3,
               subtract = subtract, dot.mov = dot)
i4 <- build_fa(main.rot = rot2, trap.rot = rot1,
               subtract = subtract, dot.mov = dot)
i5 <- build_fa(main.rot = rot3, trap.rot = rot2,
               subtract = subtract, dot.mov = dot)
i6 <- build_fa(main.rot = rot3, trap.rot = rot1,
               subtract = subtract, dot.mov = dot)
i7 <- build_fa(isomorphs = 2, a.main = 1:3, mirror = mirror,
               trap.rot = rot1, subtract = subtract, dot.mov = dot)
i8 <- build_fa(isomorphs = 2, a.main = 4:6, mirror = mirror,
               trap.rot = rot2, subtract = subtract, dot.mov = dot)
i9 <- build_fa(isomorphs = 2, a.main = c(7, 8), mirror = mirror,
               trap.rot = rot3, subtract = subtract, dot.mov = dot)

# Create 9 folders with names: "i1", …, "i9" in your PC.

# Generating an object with the route to all these folders:
route <- "C:/Desktop"   # You might want to provide another route.

# Plotting the information and saving it into the folders:
for (j in 1:9) {
  plot_fa(get(paste0("i", j)),
          directory = paste0(route, "/i", j), language.dir = "E")}
```

One more step will fully answer the researchers' requests. They solicited separate files for each basic shape of items. This can be done by setting plot mode to "C" as follows:

```
for (j in 1:9) {
  plot_fa(get(paste0("i", j)),
          directory = paste0(route, "/i", j),
          language.dir = "E",
          mode = "C")
}
```

Test instructions

Initial instructions are always necessary in order for testees to clear all potential doubts and to start the test with equal performance opportunities. This makes the test fair for all participants and it helps providing data with the required quality. It is always recommended to be specific about what participants will have to do during the test, without distressing them with so many details. For your prospective test, easy examples of each general rule are welcome, and it should be at least stated that a rule might be presented alone or together with other rule(s) in the same item. It is convenient to avoid technical terms like 'rules' or 'analogy' by replacing them with commonly understandable sentences like 'changes between the above-seen shapes' or 'the same change seen before should also occur here to complete the missing shape'. Finally, it is recommended to recollect sociodemographic data, as well as to inform the testees about the anonymity of responses and their voluntary collaboration if appropriate. The following instructions are a draft we used for online testing:

The exercises you will solve consist of two parts. In the [upper / left] part, a matrix with three shapes is presented. A fourth shape is missing, and the space that it is supposed to occupy has been filled with a question mark. In the [lower / right] part, eight options of shapes are shown, and you will have to select the missing shape out of these options by [clicking on / marking] it. If you think that the missing shape is not among the options, then choose the option 'No answer is correct'. If the exercise is so difficult that you cannot find a proper answer, then choose the option 'I don't know'. Throughout the exercises, you will find different types of changes between the shapes of the matrix, either from left to right (thus in the rows) or top down (thus in the columns). It is your task to recognize these changes and to choose, out of the given options, the one which fits in the place with the question mark. Please do not take notes while doing the test. For this task it is particularly important that you work alone and concentrate. Avoid distractions, for example from other people or media. On the next pages you will be able to work through a series of practice items.

Practice Item 1. Focus on the [upper / left] matrix. If you see the first row, you will find that the main part of the left shape makes a …° rotation (counter)clockwise to become the right shape, while both shapes are similar in the rest of their properties. As for the second row, the same part of the left shape should also rotate …° (counter)clockwise to obtain the missing shape, while these two shapes share the rest of their properties as well. So, what would be the right answer here?

Practice Item 2. Focus on the [upper / left] matrix. If you see the first row, you will find that the main part of the left shape is mirrored to become the right shape, while both shapes are similar in the rest of their properties. As for the second row, the left shape should also be mirrored in the same way to obtain the missing shape, while these two shapes share the rest of their properties as well. So, what would be the right answer here?

Practice Item 3. In the first row of this matrix, the little element that can be seen outside the circle of the left shape makes a …° rotation (counter)clockwise to become the right shape, while both shapes are similar in the rest of their properties. In the second row, the same little element of the left shape should also rotate …° (counter)clockwise to obtain the missing shape, while these two shapes share the rest of their properties as well. So, what would be the right answer here?

Practice Item 4. In the first row of this matrix, an internal line of the left shape is subtracted to become the right shape, while both shapes are similar in the rest of their properties. In the second row, the same internal line should be subtracted from the left shape to obtain the missing shape, while these two shapes share the rest of their properties as well. So, what would be the right answer here?

Practice Item 5. In the first row of this matrix, the black dot of the left shape moves towards … corner(s) in a certain direction to become the right shape, while both shapes are similar in the rest of their properties. The same movement in the same direction should be done from the left shape of the second row to obtain the missing shape, while these two shapes share the rest of their properties as well. So, what would be the right answer here?

You will now complete a series of exercises. You will have to guess which of the above described changes are active for a given exercise. It can also happen that more than one of these changes is present. For example, it is possible that the main part of the shape is rotated and, at the same time, a line is subtracted from it. It is important that you recognize all changes present between the shapes, so that you can solve the task appropriately. Please, try not to take so much time for the task and work accurately. Good luck and thank you for your collaboration.

Scoring

Before scoring, it must be noted that two important pieces of information about the items should have been saved in advance, namely the right answers and the rules applied. This information is provided in the CSV file described for the `plot.fa` function, and it is also available as output inside R when running `plot.fa`. Once the data has been recollected, responses should be converted to binary values for the cases of right (1) and wrong (0) answers respectively. Then the total score of a given test taker is simply calculated as the sum of all binary values belonging to that person. Validity and reliability assessments should be performed before any interpretation of results takes place.
