# Supplementary figures and images for: Automatic Generation of Figural Analogies With the IMak Package
Source: Front Psychol. 2018 Aug 6;9:1286. doi: 10.3389/fpsyg.2018.01286 (PMC6087760; doi:10.3389/fpsyg.2018.01286)

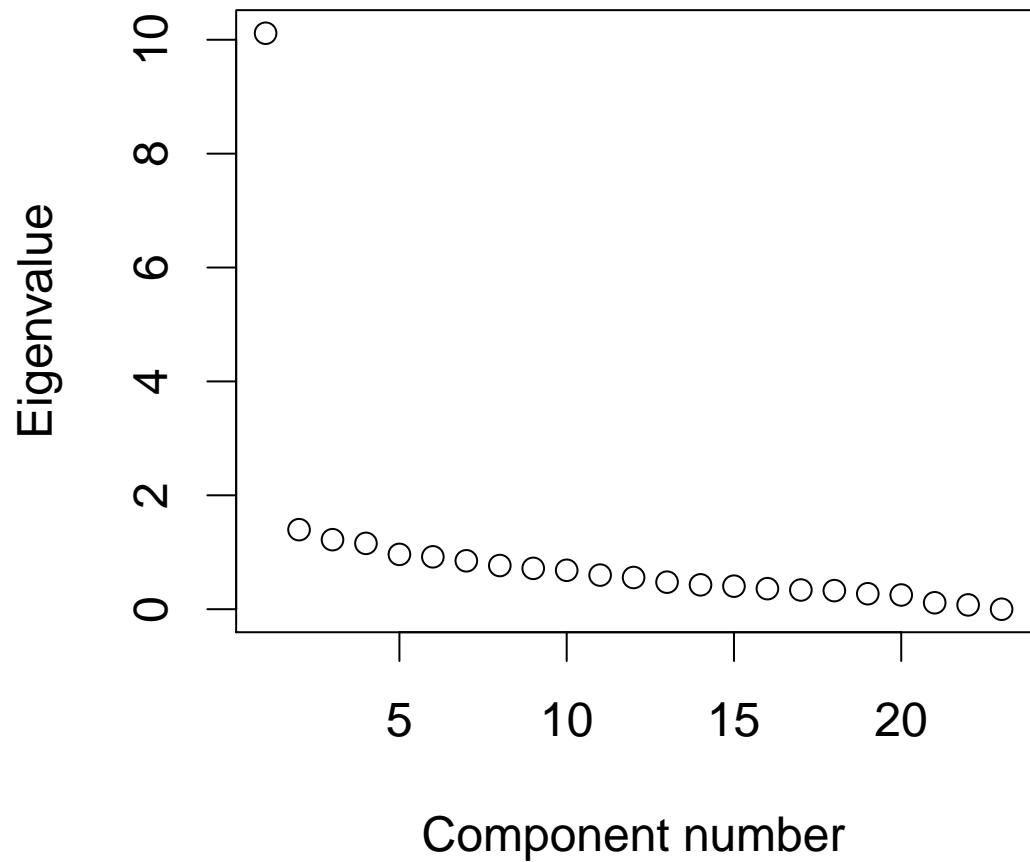

Supplement: Supplementary file 1 [file Data_Sheet_1.ZIP › Scree Plot of the current data.pdf]
